# Supplementary material for: A bioinspired self-powered optical tactile sensing system with ultrahigh sensitivity and ultralow detection limit
Source: Nat Commun. 2025 Nov 26;16:11668. doi: 10.1038/s41467-025-66792-8 (PMC12749114; doi:10.1038/s41467-025-66792-8)
Supplement: Supplementary file 1 — Supplementary Information [file 41467_2025_66792_MOESM1_ESM.pdf]

Supplementary Materials for

A bioinspired self-powered optical tactile sensing system with ultrahigh sensitivity and ultralow detection limit

*Tingting Hou[1,2], Chaojie Chen[1,2], Ru Guo[1,2], Shaoshuai He[2], Yunlong Zi[1,2,3]**

[1] Department of Mechanical and Automation Engineering, The Chinese University of Hong Kong, Shatin, N.T. Hong Kong, China

[2] Thrust of Sustainable Energy and Environment, The Hong Kong University of Science and Technology (Guangzhou), Nansha, Guangzhou, Guangdong, 511400, China

[3] Division of Integrative Systems and Design, Hong Kong University of Science and Technology, Clear Water Bay, Kowloon, Hong Kong, China

Corresponding author: * E-mail: ylzi@hkust-gz.edu.cn (Y.Z.)

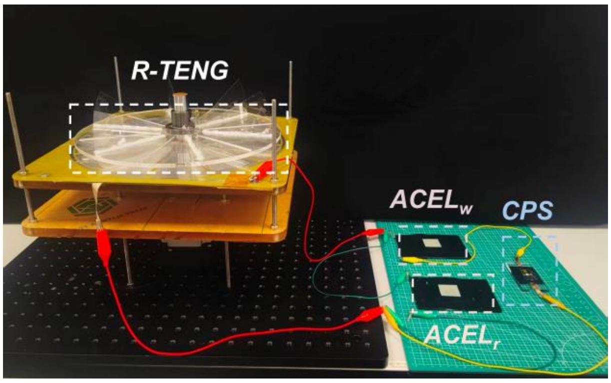

Supplementary Fig. 1. The physical photograph of the interconnections among the SOTS.

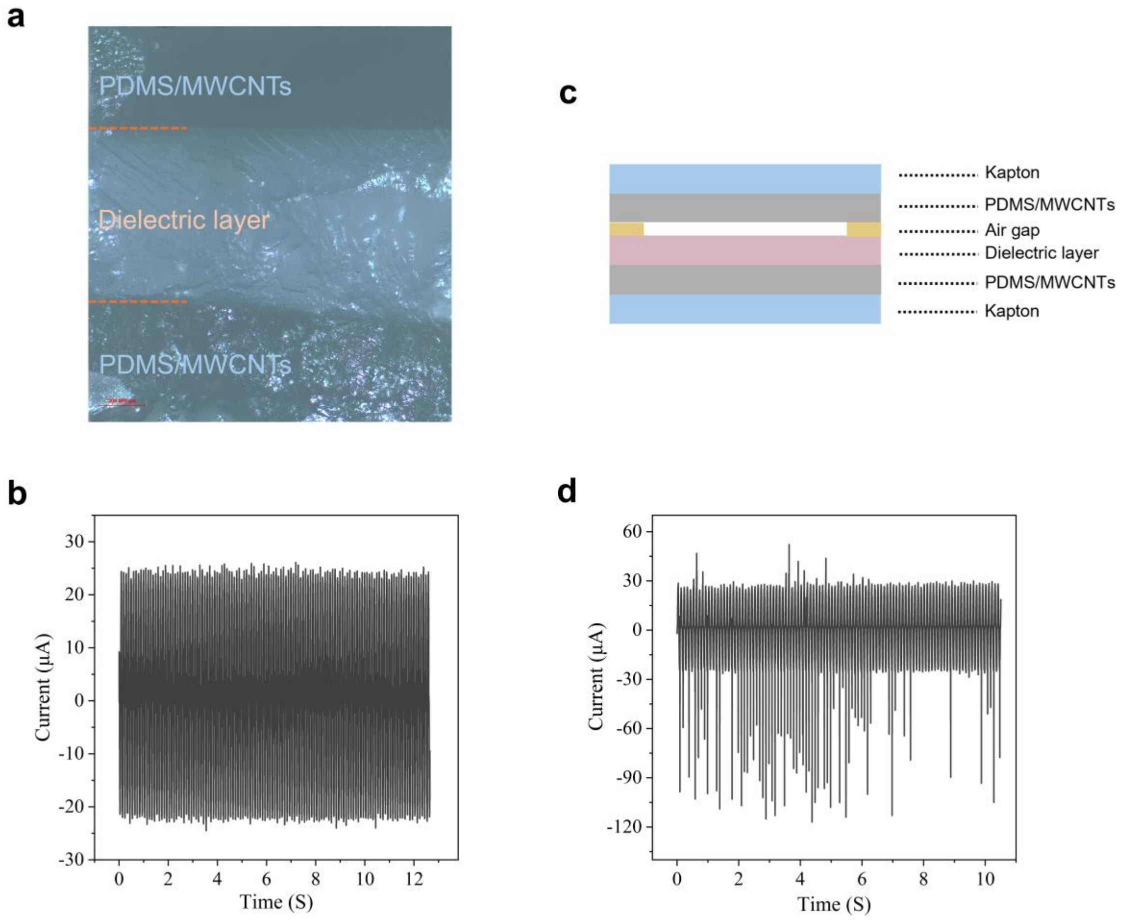

Supplementary Fig. 2. The importance of tight connections between layers in CPS. **a** The optical microscope image indicates tight contact between the layers. **b** CPS with tight structures exhibit stable current output in the R-TENG circuit. **c** The schematic diagram illustrates the presence of air gaps within the CPS structure. **d** The structure shown in **c** demonstrates a corresponding current output in the R-TENG circuit, exhibiting a significant breakdown phenomenon.

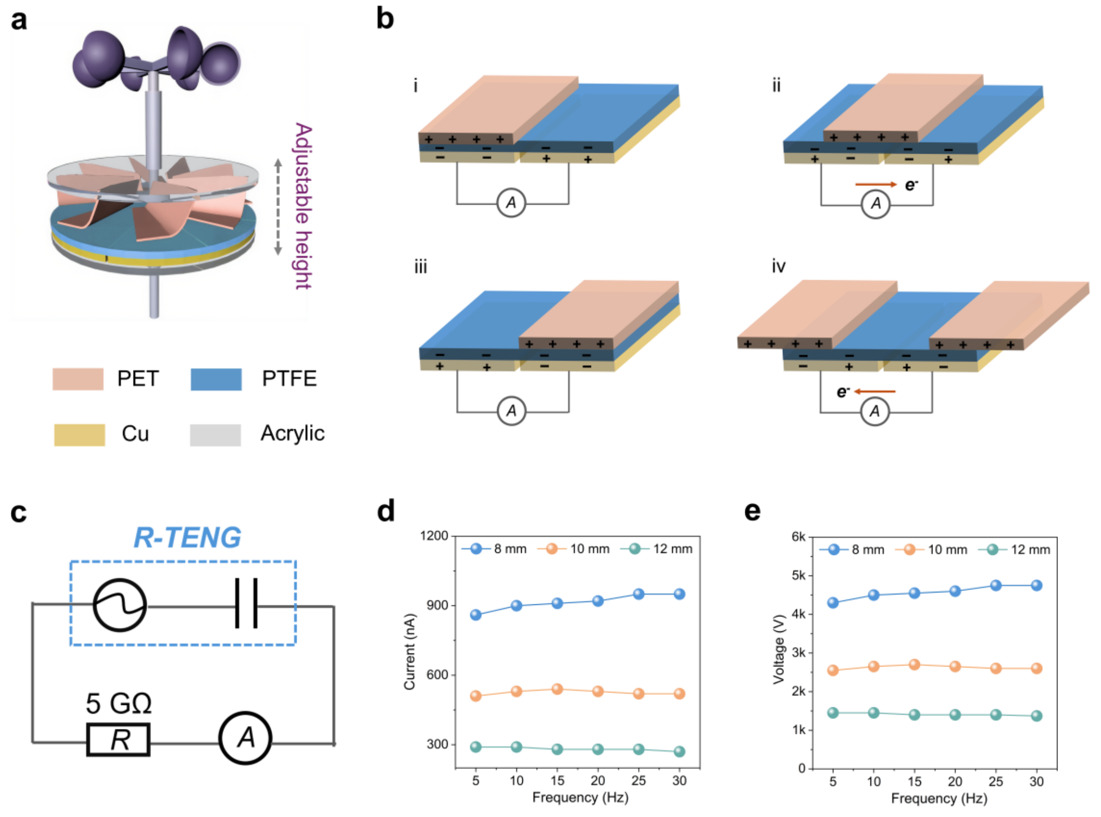

Supplementary Fig. 3. R-TENG serves as a high-voltage AC power source within the STOS system. a The structure and materials of the R-TENG allow for voltage output regulation by adjusting the upper rotator. **b** Working principles of R-TENG based on triboelectric effect and electrostatic induction. **c** The measurement principle of voltage output of R-TENG. **d** Current output generated by different rotator heights. **e** The corresponding voltage output generated by different rotator heights.

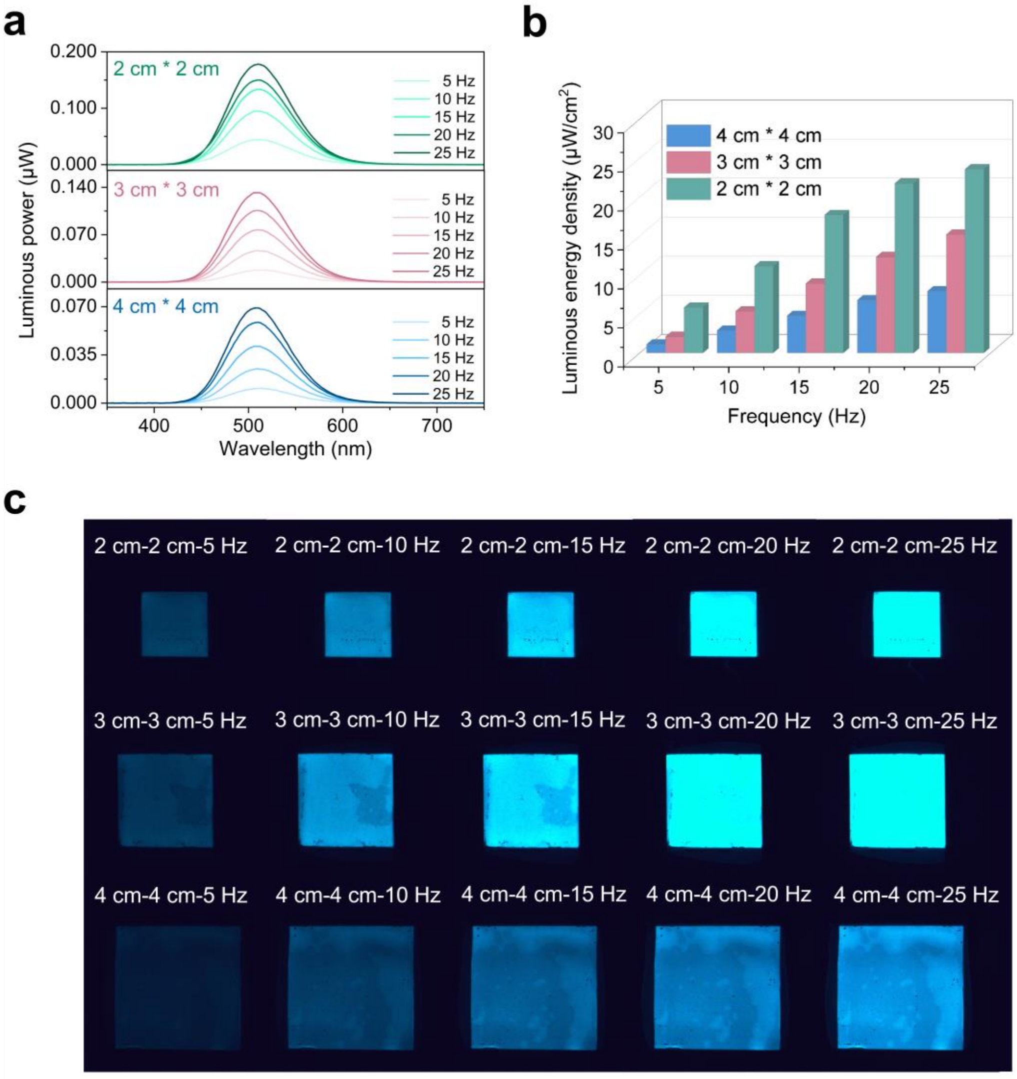

Supplementary Fig. 4. The optical performance of ACEL units with varied active areas under different frequencies. a Luminous power. **b** luminous energy density, and **c** visual images.

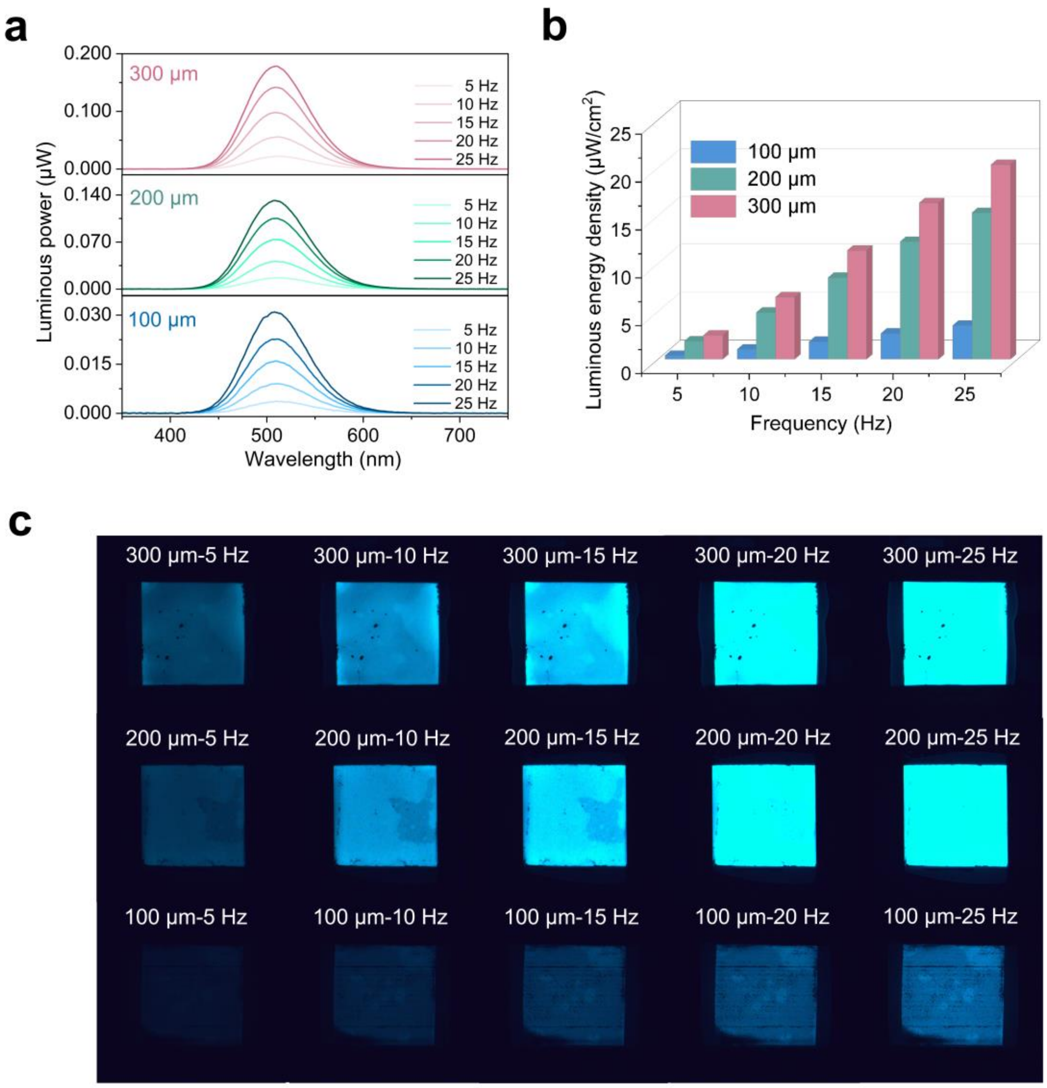

Supplementary Fig. 5. The optical performance of ACEL units with varied dielectric thickness under different frequencies. a Luminous power. **b** luminous energy density, and **c** visual images.

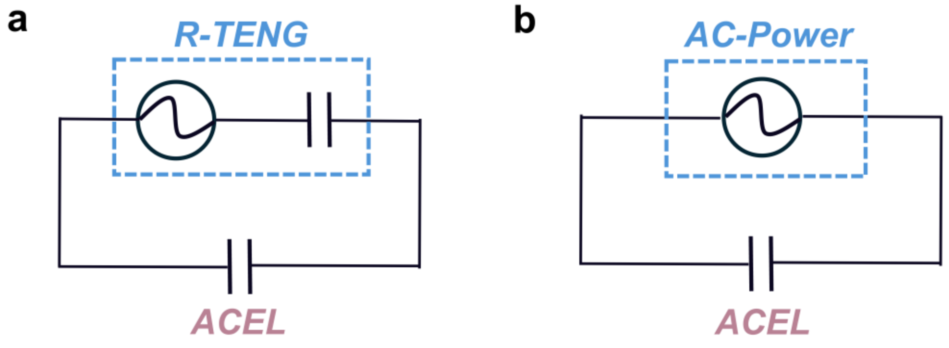

Supplementary Fig. 6. Equivalent circuit of the ACEL unit driven by R-TENG **a** and traditional AC-power **b**.

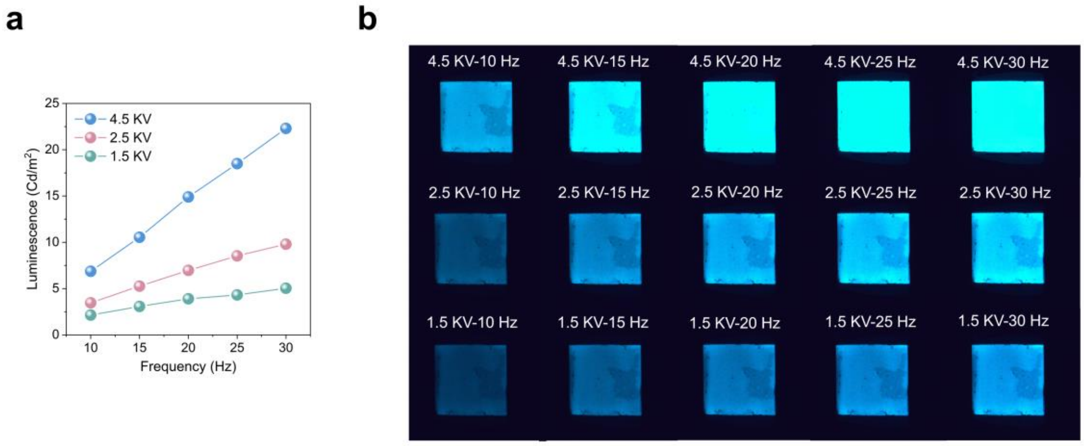

Supplementary Fig. 7. The optical performance of ACEL units with varied voltage under different frequencies. a Luminescence. **b** visual images.

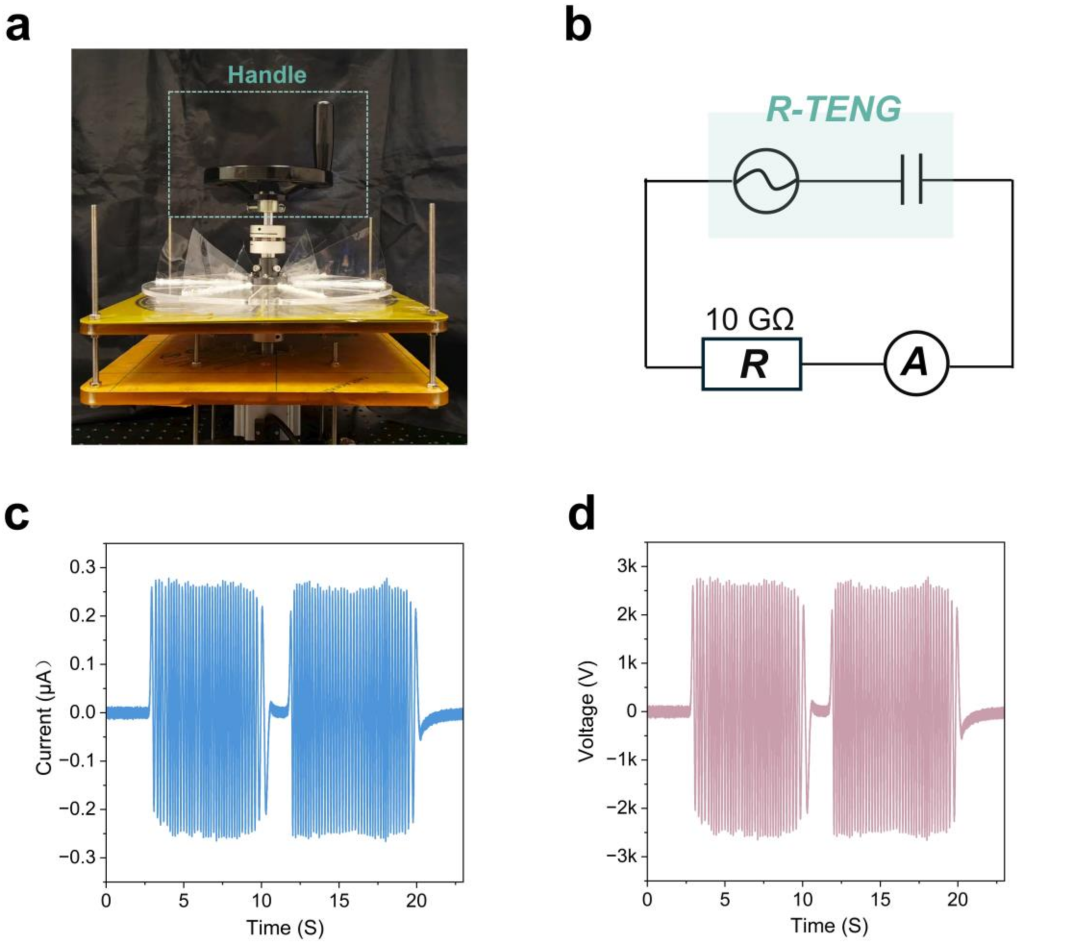

Supplementary Fig. 8. The hand-cranked R-TENG. a The photograph of the R-TENG prototype with an integrated handle on the rotor. **b** The measurement principle of voltage output of R-TENG. **c** The current output. **d** The voltage output.

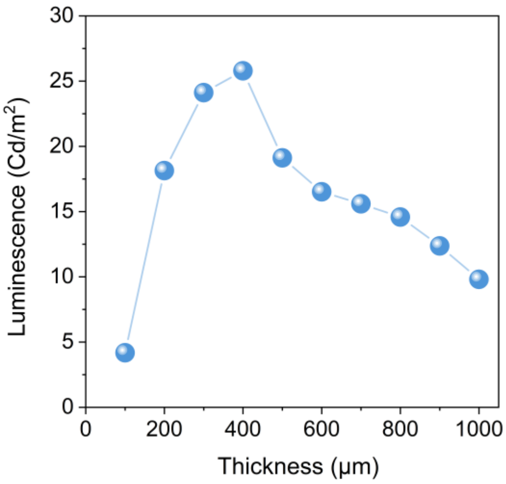

Supplementary Fig. 9. The ACEL device, connected in series with the R-TENG, exhibits luminance variations as a function of its thickness.

The luminance of the ACEL device is primarily determined by the electric field, as defined by

$$E_{ACEL} = \frac{V_{ACEL}}{d} \tag{1}$$

When the ACEL device is connected in series with the R-TENG, an increase in the dielectric thickness (d) also leads to a rise in voltage drop (V_{ACEL}). Therefore, the change in E_{ACEL} primarily depends on the relative magnitudes of these variations. The results indicate the presence of an inflection point at a thickness of 400 μm. When $d < 400$ μm, the enhancement in luminance is primarily governed by the increased V_{ACEL} resulting from its reduced capacitance, which leads to a net increase in E_{ACEL}. Conversely, when $d > 400$ μm, the reduction in E_{ACEL} due to the increased thickness becomes the dominant factor, resulting in a decrease in luminance.

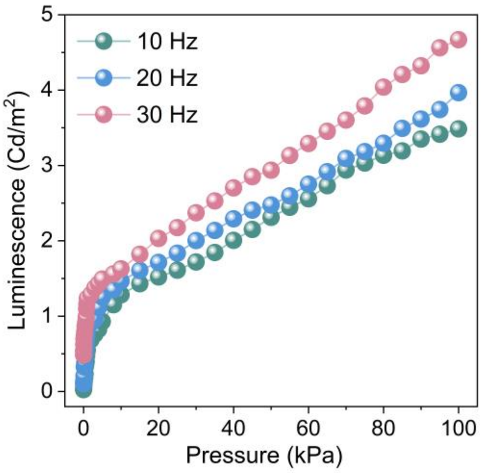

Supplementary Fig. 10. The actual luminance of the ACEL unit within SOTS.

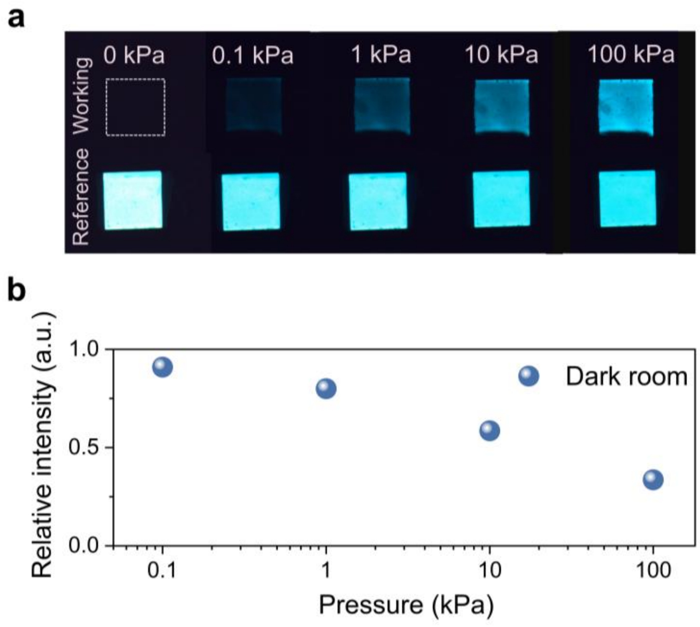

Supplementary Fig. 11. The luminance variations **a** and their relative intensity profiles **b** of the working and reference ACEL units under varied loaded pressures in darkroom.

Here, the relative intensity (ΔL) is defined as:

$$\Delta L = \frac{L_R - L_W}{L_R} \tag{2}$$

where L_R and L_W are the light intensities of reference ACEL and working ACEL, respectively. The light intensity of ACEL is captured by CCD camera and extracted the sum of pixel values via Matlab code.

The custom-developed algorithm is used to monitor real-time tactile-optical conversion processes. Upon capturing optical videos of the working and reference ACEL units with a CCD camera, the images are promptly transmitted to a laptop. The self-developed algorithm is then employed to delineate the luminous regions of both ACEL units and calculate the respective sums of their luminous pixels (L_R and L_W) based on RGB values. Hence, the relative light intensity can be obtained followed by execution of the relevant judgment procedure. Specifically, a threshold of relative intensity (L_{TH}) was established, corresponding to the level of tactile pressure that may compromise the structural

integrity of grasped object. Therefore, when $\Delta L \geq L_{TH}$, it indicates that the contact between the robotic hand and the grasped object remains within a safe range. Conversely, if $\Delta L < L_{TH}$, it suggests that the force applied by the robotic hand poses a risk of damaging the object, thus necessitating an immediate reduction in the applied pressure. The specific process flow of the algorithm is detailed in the flowchart below:

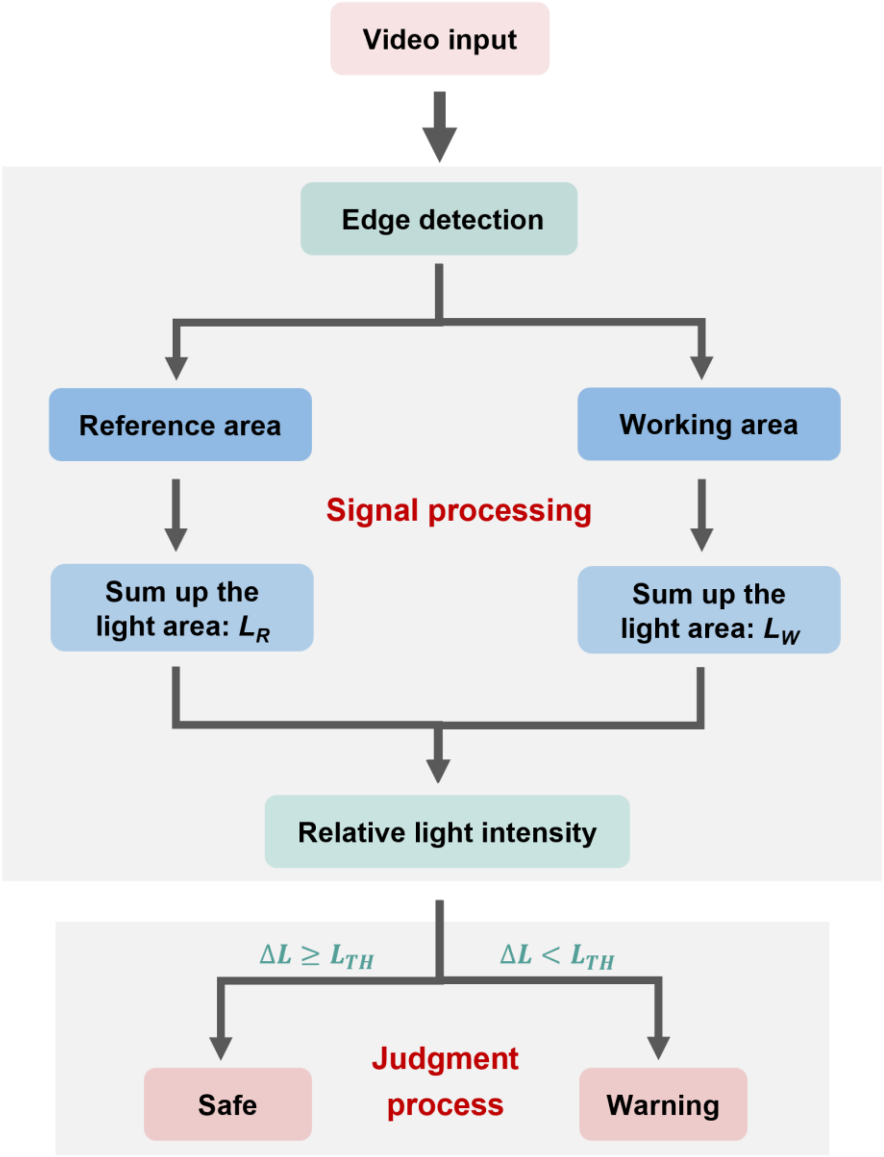

Supplementary Fig. 12. Process flow of the custom-developed algorithm for monitoring real-time tactile-optical conversion.

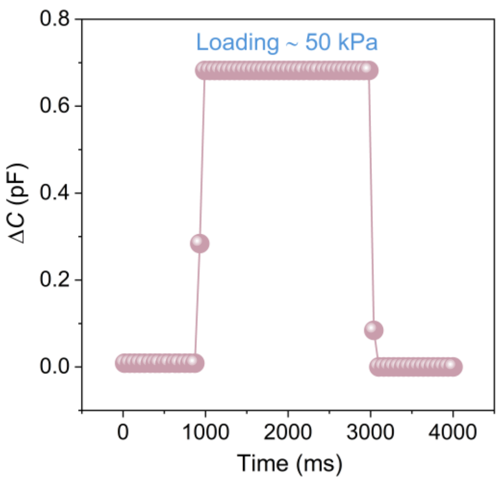

Supplementary Fig. 13. Dynamic response time of the CPS under a load of 50 kPa.

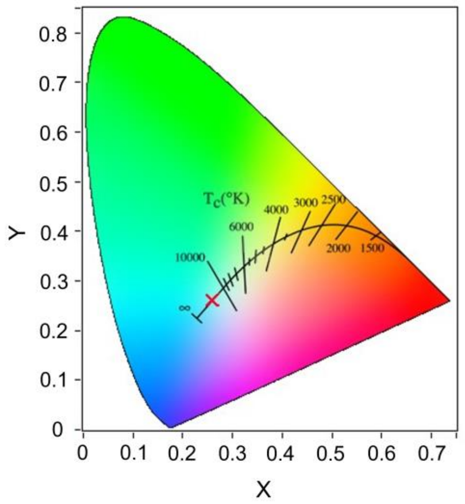

Supplementary Fig. 14. The CIE chromaticity diagram of the emitted light.

Active area (cm^2)	Dielectric thickness (μm)	Capacitance (pF)
2×2	200	238
3×3	200	437
4×4	200	717
3×3	100	832
3×3	300	278

Supplementary Table 1. The capacitance of ACEL units with different sizes.
