# Supplementary material for: A bioinspired self-powered optical tactile sensing system with ultrahigh sensitivity and ultralow detection limit
Source: Nat Commun. 2025 Nov 26;16:11668. doi: 10.1038/s41467-025-66792-8 (PMC12749114; doi:10.1038/s41467-025-66792-8)
Supplement: Supplementary file 2 — Description of Additional Supplementary Files [file 41467_2025_66792_MOESM2_ESM.pdf]

Description of Additional Supplementary Files

Supplementary Movie 1: The response of brightness to varying pressure under different frequency input.

Supplementary Movie 2: Visualized precise tactile perception enabled by SOTS.
